# Amazonian *Anopheles* with low numbers of oocysts transmit *Plasmodium vivax* sporozoites during a blood meal

**DOI:** 10.1038/s41598-022-24058-z

**Published:** 2022-11-14

**Authors:** Jordam William Pereira-Silva, Keillen Monick Martins-Campos, José Vicente Ferreira-Neto, Marcus Vinicius Guimarães Lacerda, Felipe Arley Costa Pessoa, Claudia María Ríos-Velásquez

**Affiliations:** 1 Laboratório de Ecologia de Doenças Transmissíveis na Amazônia, Instituto Leônidas e Maria Deane - FIOCRUZ Amazônia, Fundação Oswaldo Cruz - FIOCRUZ, Rua Teresina, 476, Adrianópolis, Manaus, Amazonas CEP: 69.057-070 Brasil; 2 Laboratório de Diagnóstico e Controle de Doenças Infecciosas na Amazônia, Instituto Leônidas e Maria Deane, Fundação Oswaldo Cruz - FIOCRUZ, Manaus, Amazonas Brasil; 3 Fundação de Medicina Tropical Dr. Heitor Vieira Dourado, Manaus, Amazonas Brasil; 4 Programa de Pós-Graduação em Medicina Tropical, Escola Superior de Ciências da Saúde, Universidade do Estado do Amazonas, Manaus, Brasil; 5 Programa de Pós-Graduação em Entomologia, Instituto Nacional de Pesquisas da Amazônia, Manaus, Amazonas Brasil

**Keywords:** Entomology, Infectious diseases, Malaria

## Abstract

*Anopheles darlingi* is the main malarial vector in the Brazilian Amazon region. *An. nuneztovari* s.l., *An. triannulatus* s.l., *An. evansae*, and *An. benarrochi* s.l. do not have a defined role as malarial vectors, although they have been found to be naturally infected with *Plasmodium vivax*, and some develop oocysts. In this study, we evaluated the importance of low numbers of oocysts in sporozoite salivary gland invasion and transmission. Field-collected mosquitoes were experimentally infected with *P. vivax*. The infection rates and oocyst and sporozoite infection intensities were evaluated and compared with those of *An. aquasalis*. We found the highest number of oocysts in *An. darlingi* (mean = 39.47) and the lowest in *An. nuneztovari* s.l. (mean = 2). The highest number of sporozoites was observed in *An. darlingi* (mean = 610) and lowest in *An. benarrochi* s.l. (mean = 30). *Plasmodium vivax* DNA was detected in the saliva of all mosquito species after a blood meal. Regardless of the number of oocysts, all species transmitted sporozoites during blood meals. Considering the abundance of these mosquitoes and transmission of sporozoites, it is logical to assume that *An. nuneztovari* s.l. and *An. triannulatus* s.l. are involved in the transmission of *P. vivax*.

## Introduction

*Plasmodium vivax* is the second most common cause of malaria in the world^1^. It is transmitted to humans through the bite of anopheline mosquitoes. However, not all *Anopheles* species can transmit the parasite, as transmission depends on a combination of factors, such as vectorial competence and capacity^2,3^. To be considered a competent malarial vector, the mosquito needs to allow the parasite to complete its life cycle, beginning with the ingestion of gametocytes during a blood meal in an infected host, passing through the zygote and ookinete phases in the lumen of the midgut and oocysts in the intestinal epithelium, to the sporozoites, which, after being released into the hemocoel, migrate to the salivary gland and invade it, and finally, the inoculation of infective sporozoites during the blood meal in the vertebrate host^2,4^.

During this journey, which takes an average of 14 days depending on the *Anopheles* and *Plasmodium* species, the parasite finds the physical and immunological barriers that it must go through to survive. The first barrier that the parasite must cross is the lumen of the midgut, where hundreds of gametocytes arrive after an infected blood meal, but less than 5% develop into oocysts^5^. The second major barrier to *Plasmodium* development is the hemocoel cavity, where sporozoites are released after oocyst rupture, but only approximately 25% of them migrate to and invade the mosquito salivary glands^5^. Therefore, this complex and highly toxic journey leads to the death of some parasites and survival of others. In other words, the conditions are neither completely toxic nor completely favorable. Although it is known that this miscellany of conditions can cause a mechanism known as refractoriness, the mechanism by which some mosquitoes are able to resist infection is still unknown^6^.

The Brazilian Amazon region is home to a high diversity of mosquitoes with different epidemiological importance in malaria transmission. Although 49 species of *Anopheles* have been described in the Brazilian Amazon region, only 19 were found to be naturally infected with *Plasmodium* spp.^7,8^. *Anopheles darlingi* is an important malaria vector in the Amazon region; however, the role of other species as malaria vectors, such as *An. nuneztovari* s.l., *An. triannulatus* s.l., and *An. benarrochi* s.l., which have been found to be naturally infected with *P. vivax*, has not yet been demonstrated^9–13^. Ríos-Velásquez et al.^14^ proposed that in the Brazilian Amazon region, *An. nuneztovari* s.l. is involved in the transmission of *P. vivax* owing to the abundance of this species in the areas where *An. darlingi* is also abundant, in addition to the high intensity of infection observed in the laboratory. However, owing to the low number of oocysts, the authors reinforced the idea that *An. triannulatus* is not involved in the transmission of *P. vivax*, although this species is more abundant than *An. nuneztovari* s.l.

Therefore, it is of great interest to understand the significance of the number of oocysts in the mosquito midgut for sporozoite salivary gland invasion and *P. vivax* transmission to vertebrate hosts. In this study, five field-collected *Anopheles* species frequently recorded in peri-urban areas of the city of Manaus were used to evaluate the importance of low or high number of oocysts in sporozoite salivary gland invasion and transmission. *Anopheles aquasalis*, a vector in the coastal regions of Brazil that colonizes under laboratory conditions, has been used as a reference species.

## Results

### Field collections

At all 2.224 mosquitoes were collected and reared in laboratory to obtain F1 females. Only five species were collected during the fieldwork at the studied localities. The most abundant species were *An. triannulatus* s.l. with 996 individuals (44.78%), followed by *An. darlingi* with 467 (20.99%), *An. nuneztovari* s.l. with 366 (16.45%), *An. evansae* with 298 (13.39%), and *An. benarrochi* s.l. with 97 (4.36%).

### Mosquitoes

**Figure 1:**
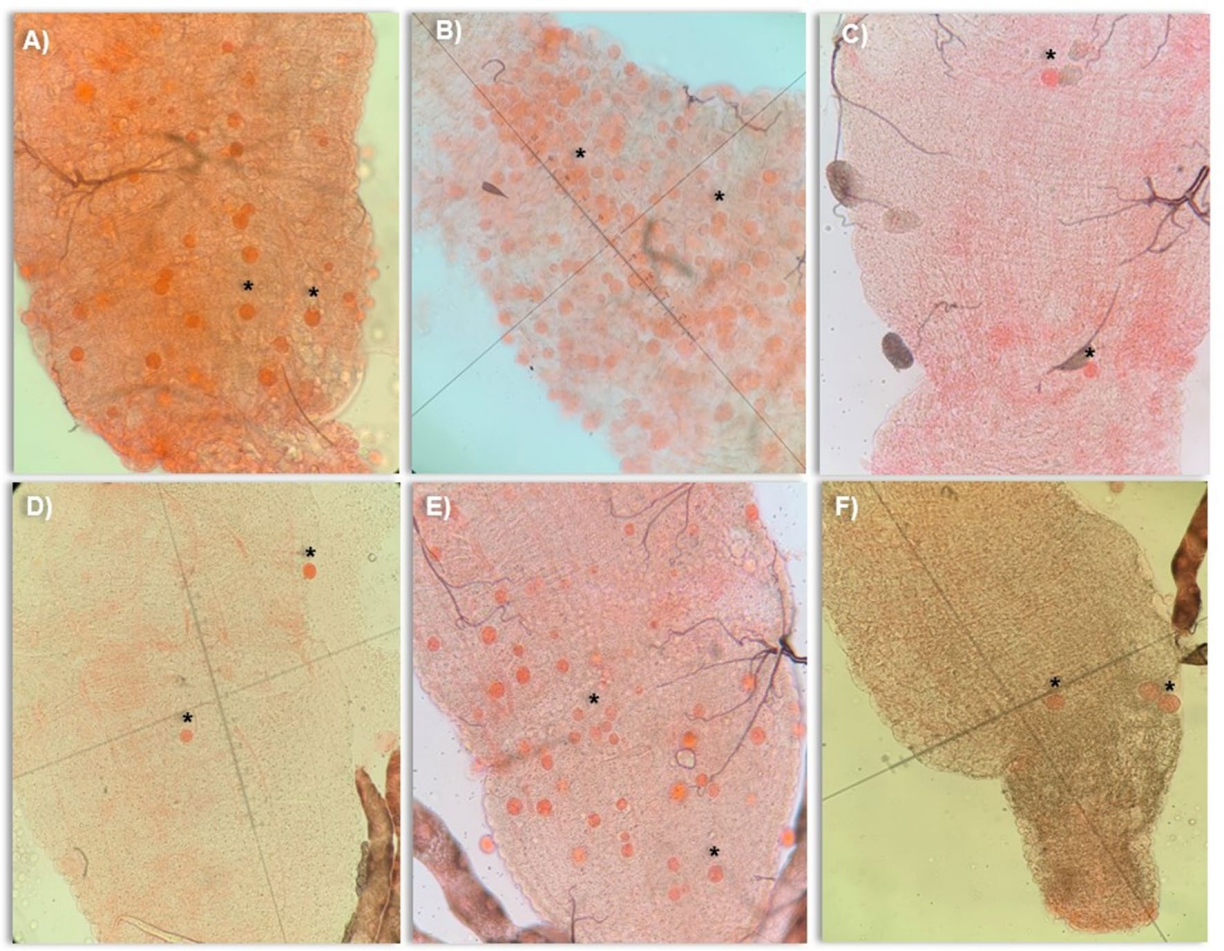
Midgut of six Amazonian *Anopheles* species with *P. vivax* oocysts that developed on the seventh day after experimental infection. (**A**) *An. aquasalis*; (**B**) *An. darlingi*; (**C**) *An. nuneztovari* s.l.; (**D**) *An. triannulatus* s.l.; (**E**) *An. evansae*; (**F**) *An. benarrochi* s.l. * = oocyst.

A total of 913 adult female mosquitoes corresponding to six F1 *Anopheles* species (*An. triannulatus* s.l.*, An. nuneztovari* s.l.*, An. evansae, An. benarrochi* s.l. *and An. darlingi*), and *An. aquasalis* were used to evaluate the susceptibility and transmission of *P. vivax* (Table 1, Fig. 1). In addition, six malaria-infected patients were included in the experimental infections using the membrane feeding assay.

**Table 1:** Susceptibility of six Amazonian *Anopheles* species fed with blood samples obtained from malaria-infected patients.

Species	Mosquitoes dissected	IR (%)	*p* value	Oocysts per mosquito (Min–Max)	*p* value	Mosquitoes dissected	Sporozoites per mosquito (Min–Max)	*p* value
*An. aquasalis* (ref)	140	76.42	–	39.18 (0–238)	–	140	390 (25–1000)	–
*An. darlingi*	50	76	0.9477	39.47 (0–218)	0.8273	50	610 (375–800)	0.3051
*An. nuneztovari* s.l	30	20	0.0003	2 (0–3)	< 0.0001	27	204 (150–357)	0.4928
*An. triannulatus* s.l	116	15.51	< 0.0001	5.16 (0–36)	< 0.0001	96	141 (25–294)	0.0089
*An. evansae*	64	7.81	< 0.0001	14 (0–45)	0.2158	40	88 (25–150)	0.0065
*An. benarrochi* s.l	20	5	‡	3 (0–3)	‡	20	30 (30–30)	‡

*Ref* reference; *IR* Infection rate; ‡: not included in the analysis.

### Infection rate

**Figure 2:**
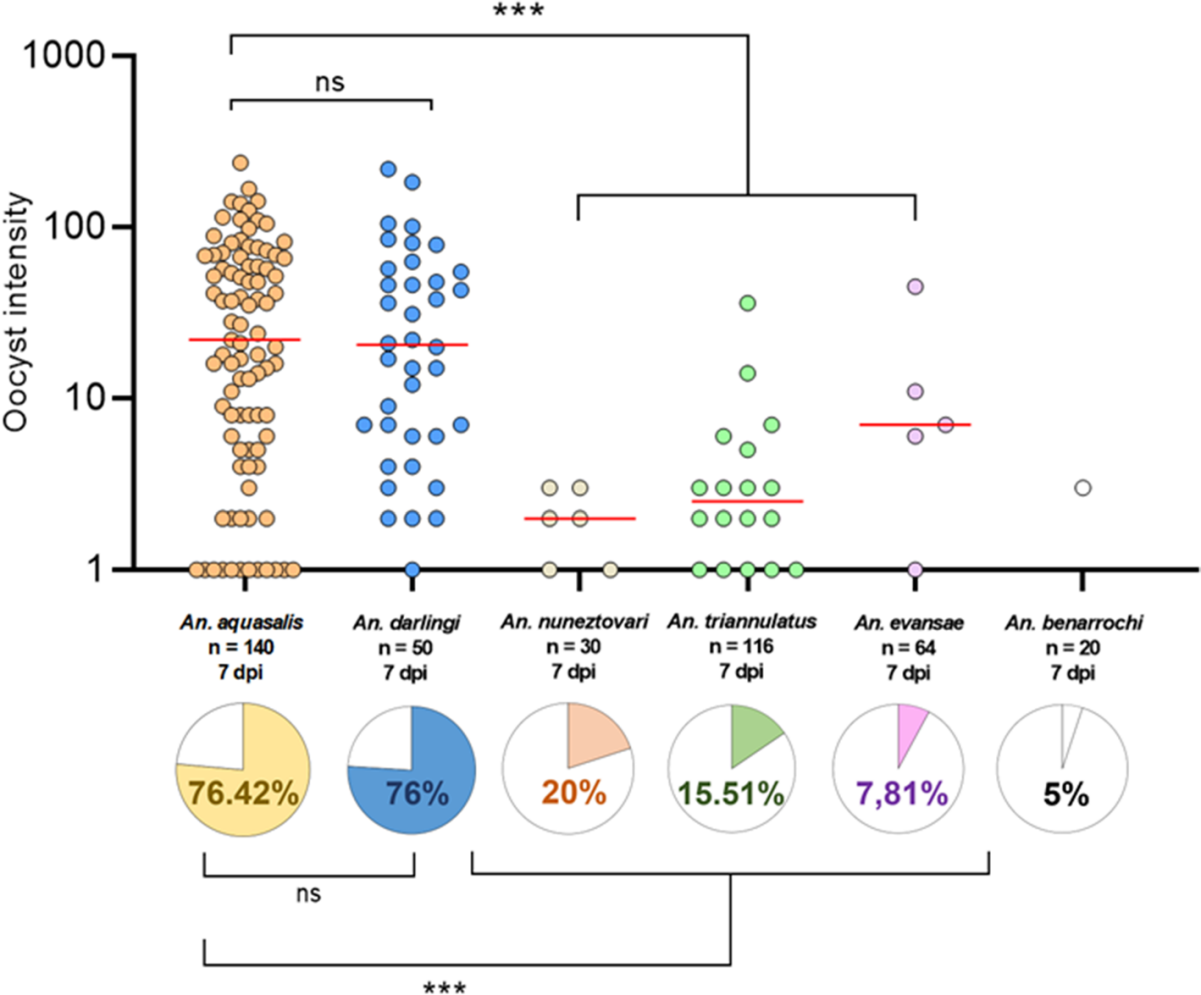
Infection rates and number of *P. vivax* oocysts in the midgut of six *Anopheles* species*.* ***p < 0.0001: Statistically significant; ns: statistically non-significant difference; dpi: days post-infection. Plotted with GraphPad Prism v.6.00 software (San Diego, CA, USA, https://www.graphpad.com/).

All *Anopheles* species were susceptible to *P. vivax* infection; however, the infection rates differed among them (Table 1, Fig. 2). *Anopheles aquasalis* had the highest infection rate (mean IR = 76.42), followed by *An. darlingi* (mean IR = 76), *An. nuneztovari* s.l. (mean IR = 20), *An. triannulatus* s.l. (mean IR = 15.51), *An. evansae* (mean IR = 7.81), and *An. benarrochi* s.l. (mean IR = 5). Compared with the reference species, *An. aquasalis*, there were no statistically significant differences in the infection rate of *An. darlingi* (*p* = 0.9477), but there was a significant difference in *An. nuneztovari* s.l. (*p* = 0.0003), *An. triannulatus* s.l. (*p* < 0.0001), and *An. evansae* (*p* < 0.0001) (Table 1, Fig. 2). Infection rates were not significantly different among *An. nuneztovari* s.l.*, An. triannulatus* s.l., and *An. evansae* (*p* = 0.1450); however, the infection rate of *An. nuneztovari* s.l. was higher.

### Intensity of oocyst infection

The infection intensity, measured as the number of oocysts per infected mosquito, varied significantly among the species studied (Table 1, Fig. 1). The highest oocyst infection intensity was observed in *An. darlingi* (mean = 39.47, median = 20.5, oocyst range = 0–218), followed by *An. aquasalis* (mean = 39.18, median = 19.5, oocyst range = 0–238), *An. evansae* (mean = 14, median = 7, oocyst range = 0–45), *An. triannulatus* s.l. (mean = 5.16, median = 2.5, oocyst range 0–36), *An. benarrochi* s.l. (mean = 3, median = 3, oocyst range = 0–3), and *An. nuneztovari* s.l. (mean = 2, median = 2, oocyst range = 0–3). When comparing the oocyst infection intensity of the studied species with that of the reference species, there was no statistically significant difference between *An. darlingi* (*p* = 0.8273) and *An. evansae* (*p* = 0.2158); however, there was a significant difference for *An. triannulatus* s.l. (*p* < 0.0001) and *An. nuneztovari* s.l. (*p* < 0.0001).

The infection intensity varied between species and was lower than that of *An. aquasalis. Anopheles nuneztovari* s.l. had 19.59-fold fewer oocysts than *An. aquasalis*, followed by *An. benarrochi* s.l. with 13.06-fold fewer oocysts, *An. triannulatus* s.l. with 7.59-fold fewer oocysts, and *An. evansae* with 2.79-fold fewer oocysts; however, *An. darlingi* presented 0.98-fold more oocysts than did *An. aquasalis*.

### Sporozoite infection

**Figure 3:**
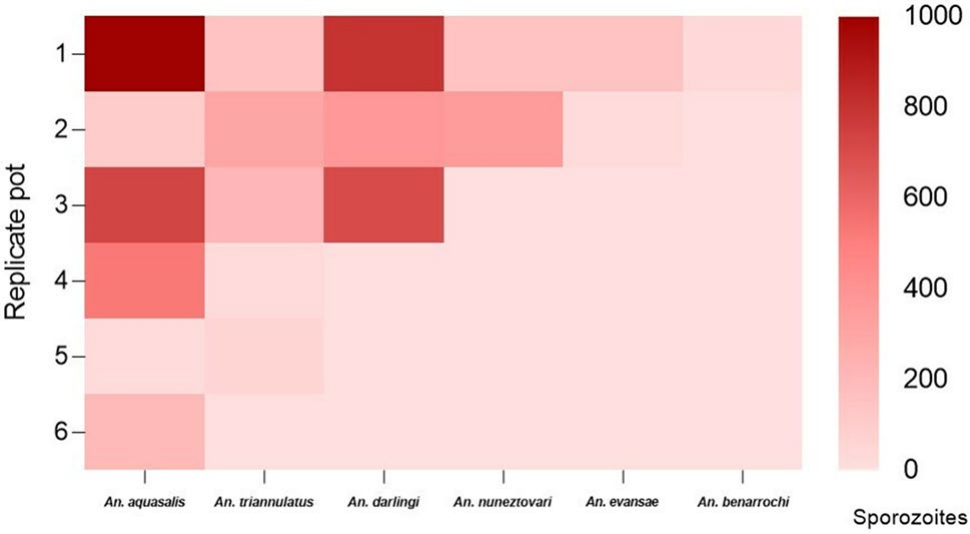
Heat map showing the relative amounts of *P. vivax* sporozoites in the salivary glands of *Anopheles* species. Mean number of sporozoites per mosquito. Each row shows the sporozoite quantification per replicate and each column shows the sporozoite number arranged from top to bottom according to species. Plotted with GraphPad Prism v.6.00 software (San Diego, CA, USA, https://www.graphpad.com/).

The salivary glands of all studied species were infected by *P. vivax* sporozoites; however, the number of sporozoites varied among them (Table 1, Fig. 3). A higher intensity of sporozoites per mosquito was observed in *An. darlingi* (mean = 610, median = 700, range = 375–800), followed by *An. aquasalis* (mean = 390, median = 200, range = 25–1.000), *An. nuneztovari* s.l. (mean = 204, median = 254, range = 150–357), *An. triannulatus* s.l. (mean = 141, median = 150, range = 25–294), *An. evansae* (mean = 88, median = 88, range = 25–150), and *An. benarrochi* s.l. (mean = 30, median = − 30, range = 30–30) (Table 1). Comparing the sporozoite infection intensity of the evaluated species with that of the reference species, no statistically significant difference was observed in *An. darlingi* (*p* = 0.3051) and *An. nuneztovari* s.l. (*p* = 0.4928); however, it was significantly different for *An. triannulatus* s.l. (*p* = 0.0089) and *An. evansae* (*p* = 0.0067).

**Figure 4:**
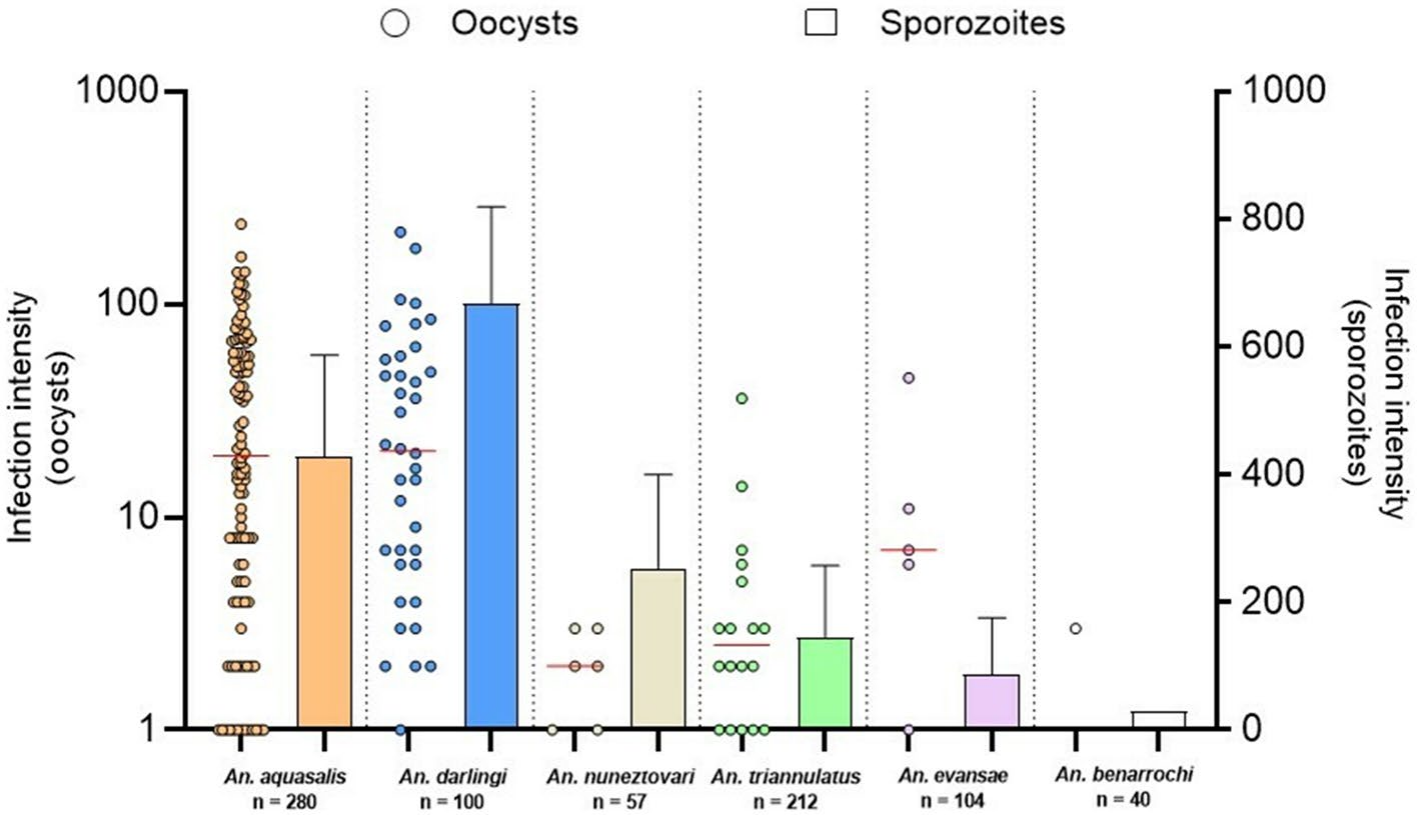
Comparison between the number of sporozoites and oocysts in six species of *Anopheles* infected with *P. vivax*. Plotted with GraphPad Prism v.6.00 software (San Diego, CA, USA, https://www.graphpad.com/).

Although *An. nuneztovari* s.l., *An. triannulatus* s.l., *An. evansae,* and *An. benarrochi* s.l. had few oocysts, they all had salivary glands invaded by *P. vivax* sporozoites at 14 dpi (Fig. 4). Compared with the reference species *An. benarrochi* s.l. had 12.97-fold fewer sporozoites, followed by *An. evansae* with 4.44-fold fewer sporozoites, *An. triannulatus* s.l. with 2.76-fold fewer sporozoites, and *An. nuneztovari* s.l. with 1.91-fold fewer sporozoites. *Anopheles darlingi* had a 1.56-fold higher number of sporozoites than did *An. aquasalis.*

Correlation analyses between oocysts and sporozoites revealed a moderate to strong correlation among *An. aquasalis* (r = 0.536, *p* = 0.215), *An. darlingi* (r = 0.968, *p* = 0.159), *An. triannulatus* s.l. (r = 0.231, *p* = 0.659), *An. nuneztovari* s.l. (r = 1, *p* < 0.001), and *An. evansae* (r = 1, *p* < 0.001). Because of the low number of individuals available for experimentation, analyzing the correlations for *An. benarrochi* s.l. was not possible.

### Detection of *P. vivax*

**Figure 5:**
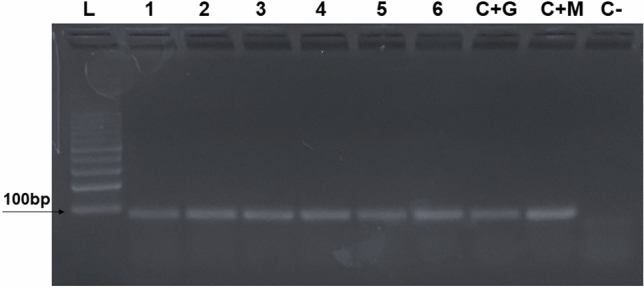
Polymerase chain reaction for detection of *P. vivax* in the saliva of six species of *Anopheles.* (L) DNA Ladder; (**1**) *An*. *triannulatus*; (**2**) *An*. *nuneztovari*; (**3**) *An*. *benarrochi*; (**4**) *An*. *evansae*; (**5**) *An*. *darlingi*; (**6**) *An*. *aquasalis*; (**C + G**) Positive control (salivary gland + *P. vivax*); (**C + M**) Positive control (midgut + *P. vivax*); (**C-**) Negative control. Original gels are presented in Supplementary Fig. 1.

*P. vivax* DNA was detected in the saliva of all the *Anopheles* species studied after the salivation test. A DNA fragment of 100 bp was detected, corresponding to the Pv18S gene pattern (Fig. 5). Original gels are presented in Supplementary Fig. 1.

## Discussion

The diversity of *Anopheles* species is high in the Brazilian Amazon region, with approximately 49 species recorded to date, 19 of which have been found infected with human *Plasmodium* species^7,8,15^. *Anopheles darlingi* is an important malaria vector in the Amazon region^16^. Other species including *An. albitarsis* s.l*., An. deaneorum, An. oswaldoi, An. mediopunctatus* s.l.*, An. triannulatus* s.l.*, An. nuneztovari* s.l., and *An. marajoara* are potentially regional vectors because they are commonly collected at high densities in the Amazon region, show anthropophilic behavior, and have been found naturally infected with human *Plasmodium* species^17^. However, the vector status of these species is yet to be elucidated.

In this study, we selected six *Anopheles* species to evaluate the significance of having either few or many oocysts from the viewpoint of sporozoite salivary gland infection and transmission. The species selected were *An. aquasalis*, which was used as a reference species because it is colonized under laboratory conditions, and the other five species (*An. darlingi, An. nuneztovari* s.l.*, An. triannulatus* s.l.*, An. benarrochi* s.l., and *An. evansae*) were most frequently collected in peri-urban areas of the city of Manaus. Mosquitoes were fed on blood samples obtained from malaria-infected patients using a membrane feeding assay, which although an experimental method, has been shown to be suitable for mosquito susceptibility studies^14,18–20^.

*An. darlingi* showed high infection rates and oocyst intensities similar to those of *An. aquasalis*, but higher than those reported by Rios-Velásquez et al.^14^. Similar oocyst and sporozoite production rates were observed in field populations of *An. darlingi* from the Peruvian Amazon, with an oocyst infection intensity of 13 (range of 1–93) and a sporozoite yield per mosquito of 1080 (range, 170–7380)^21^.

*Anopheles aquasalis* and *An. darlingi* are considered important malaria vectors in different regions of the Americas, including the Amazon Region^3,7^; therefore, they are expected to be highly susceptible to human *Plasmodium* species such as *P. vivax*. Variations in infection rates and intensities are related to human hosts, parasites, and mosquito vector factors, such as genetics, immunity, biology, and ecology, which can vary among individuals and populations^2,22,23^.

*Anopheles nuneztovari* s.l. and *An. triannulatus* s.l. showed substantially lower infection rates and oocyst and sporozoite intensities than did *An. aquasalis* or *An. darlingi* but similar to those observed by Rios-Velasquez et al.^14^. These two species are considered important malaria vectors in some places in Latin America^16^; however, their vectorial competence in the Amazon region has not been elucidated and they are considered as occasional vector in some localities. *Anopheles triannulatus* s.l. had the fourth highest infection rate (15.51%) and oocyst intensity (5.16). Furthermore, an average of 141 sporozoites were counted per salivary gland pair. In Brazil, *An. triannulatus* s.l. has been found to be naturally infected by *P. vivax* and *P. falciparum*, with infection rates between 0.23 and 0.56%^15,24^. Under experimental conditions, the infection rate was 8.8%^14^. Although in Brazil *An. triannulatus* showed low susceptibility to *P. vivax* infection^14,25^, it is a vector in Colombia, Peru, and Venezuela^26–28^. *Anopheles triannulatus* s.l. has preferential zoophilic habits, and this behavior is probably the limiting factor for the transmission of human malaria. However, at high densities, it can behave as an opportunistic species, and depending on the availability of vertebrate hosts, it can play a role in malaria transmission. In the present study, these species showed attractiveness to humans, as observed in other studies^29,30^. The evidence that those two species can bite humans in addition to the susceptibility to *P. vivax* and the capacity to develop sporozoites indicates that they must be important for maintenance of malaria transmission cycles.

*Anopheles nuneztovari* s.l. showed infection rates and intensities of 20% and 2, respectively. Furthermore, an average of 204 sporozoites were counted per salivary gland pair. Although statistically different from *An. aquasalis* and *An. darlingi*, *An. nuneztovari* s.l., it was not statistically different from *An. triannulatus* s.l., *An. evansae*, or *An. benarrochi* s.l. (Table 1). The importance of vectors that are considered secondary or occasional has increased markedly over the years. This species is considered epidemiologically unimportant; however, outside of the Brazilian Amazon, *An. nuneztovari* s.l. is associated with malaria transmission in Venezuela, Colombia, and Peru^26,31,32^.

Our results showed that *An. nuneztovari* s.l and *An. triannulatus* s.l were permissive for *P. vivax* development in the midgut and salivary glands, and for the transmission of sporozoites during blood meals. Despite the lower rates of oocysts in *An. nuneztovari* s.l and *An. triannulatus* s.l., sporozoite production was higher than that observed in *An. evansae*, which had a higher mean oocyst count and a lower quantity of sporozoites.

*Anopheles evansae* and *An. benarrochi* s.l. that showed the lowest infection rates among the studied species, have not been considered potential vectors, and are found in low abundance in the field. *Anopheles benarrochi* s.l. was collected for the first time infected naturally by *P. vivax* in the Brazilian Amazon region^13^. *An. evansae* is not naturally infected by *Plasmodium* spp.^15^. Nevertheless, in this study, both species were permissive to *P. vivax* development in the midgut and salivary glands and transmitted the parasite during a blood meal.

It is important to note that *An. benarrochi* s.l. showed a lower infection rate and parasite load in the midgut and salivary glands, however, because of the low number of individuals available for experimentation, *An. benarrochi* s.l. was not included in the statistical analysis. This is the first time that *An. benarrochi* s.l. has been experimentally infected by membrane feeding assay, and these results agree with its status as “non vector.” Little is known about the epidemiological importance of *An. benarrochi* s.l. in Brazil. Klein et al.^25^ showed that this species developed *P. vivax* oocysts under laboratory conditions; however, sporozoites were not found in the salivary glands. In this study, we examined midgut oocyst formation and sporozoite transmission. Although we did not consider the vector species in the Brazilian Amazon, *An. benarrochi* s.l. is a vector for *P. vivax* and *P. falciparum* in the Peruvian Amazon^33^. *Anopheles benarrochi* s.l. is part of a species complex^34,35^ and different geographical populations probably have different susceptibilities to *Plasmodium* infection. The population density of this species is very low in the Brazilian Amazon^25,36,37^; therefore, the number of available individuals for experimentation was too low. Combining the infection rate, intensity of oocysts and sporozoites, and low population densities, it is reasonable to suggest that this species does not significantly participate in *P. vivax* transmission cycles. This type of work must be done in areas where this species is more abundant to clarify its role in *Plasmodium* transmission.

Despite the low number of oocysts observed in *An. nuneztovari* s.l., *An. triannulatus* s.l., *An. evansae*, and *An. benarrochi* s.l., their salivary glands in infected insects were found with sporozoites. A correlation was found between the number of oocysts in the midgut and the number of sporozoites in the salivary glands of the studied species. Santos et al.^38^ reported a positive correlation between the number of *P. vivax* oocysts and sporozoites in *An. darlingi* and *An. deaneorum*-infected mosquitoes. The presence of oocysts in the mosquito midgut is considered a parameter for determining the susceptibility of *Anopheles* to *Plasmodium*. These results indicate that in these Amazonian *Anopheles* species, the presence of oocysts in the midgut would lead to salivary gland invasion by sporozoites, regardless of the number of oocysts; that is, a mosquito with oocysts is potentially infectious.

Despite the low numbers of oocysts in *An. nuneztovari* s.l., *An. triannulatus* s.l., *An. evansae*, and *An. benarrochi* s.l., the resulting sporozoites were transmitted during blood meals. The salivary glands of mosquitoes are invaded by thousands of sporozoites; however, only 10–100 sporozoites are inoculated in the vertebrate host^39^. The smallest number of sporozoites (mean of 30 sporozoites per mosquito) was counted in the salivary glands of *An. benarrochi* s.l. Mosquitoes with ≤ 10 sporozoites can initiate an infection in 32% of vertebrate hosts and mosquitoes with > 1000 sporozoites in 78%^40^. In this study, we observed sporozoite motility in the salivary glands of all *Anopheles* species, demonstrating that sporozoites were viable at 14 dpi (S1 Movie). Sporozoite motility is a fundamental requirement for migration to the salivary glands, exiting the dermal tissue, and reaching the bloodstream of the vertebrate host^41,42^.

Studies on mosquito infectivity generally focused on oocysts and on blocking their development^43–45^, despite the fact that it is the sporozoites that are key to *Plasmodium* transmission. This study shows, for the first time, that in the Amazon mosquito species studied here: (a) a low number of oocysts usually translates into salivary gland invasion, and (b) if the salivary gland is invaded, the sporozoites can be transmitted during the blood meal.

The available strategies for blocking transmission only reduce the intensity of infection in mosquitoes^46,47^, but what is the significance of this from the point of view of *Plasmodium* transmission? According to our results, low numbers of oocysts are insufficient to prevent salivary gland invasion or parasite transmission. Thus, to interrupt malaria transmission, any intervention must result in the complete inhibition of oocyst formation.

We detected *P. vivax* DNA in the saliva of six *Anopheles* species, two known vector species, and four non-vector species in the Brazilian Amazon Region. These results show for the first time that *P. vivax* completes its development in *An. triannulatus* s.l., *An. nuneztovari* s.l., *An. evansae*, and *An. benarrochi* s.l. The parasite invades the intestinal epithelium and salivary glands and is expelled during bloodmeals. We showed that mosquitoes with low numbers of oocysts can transmit the parasite during a blood meal. From an epidemiological point of view, whether there are few or many oocysts does not necessarily impact sporozoite transmission. The question is: What is the role of these mosquito species in malaria transmission in the areas where *An. darlingi* is in low abundance? It could be expected that mosquito species that have infected salivary glands and are able to eject sporozoites during a blood meal could also play an important role in maintaining the number of malaria cases in the Amazon region.

## Conclusion

In this study, we showed that at least three oocysts in the midgut are sufficient for successful salivary gland invasion and *P. vivax* transmission. We conclude that populations of Amazonian *Anopheles* species with low numbers of oocysts are robust transmitters of *P. vivax*. Thus, alternative malaria elimination strategies are needed to identify and explore mechanisms that block, rather than suppress, transmission of the parasite. *Anopheles triannulatus* s.l., *An. nuneztovari* s.l., *An. evansae*, and *An. benarrochi* s.l. have high potential as malaria vectors; therefore, our results suggest that these species may act occasionally as competent vectors of *P. vivax* in nature. In the area of mosquito collection, the abundance of mosquito species changes throughout the year, similar to the substitution of species according to weather. At the same time, in these localities, malaria transmission occurred throughout the year. Our hypothesis is that in the absence of *An. darlingi*, species such *An. nuneztovari* s.l., and *An. triannulatus* s.l. helps to maintain transmission of *P. vivax.*

## Methods

### *Anopheles* collections

In total, 30 field collections were made during March 2021 to April 2022 in three sites located in the Manaus municipality, Amazonas State, Brazil. Mosquito larvae were collected in breeding sites at Puraquequara neighborhood (3°02′40.1″S 59°53′47.5″W) and Brasileirinho Roads (3°01′25.0″S 59°52′55.2″W), during the daytime from 08:00 to 12:00. Adult mosquitoes were collected biting horses, using the mouth aspirator method at twilight from 16:00 to 20:00, in a ranch located at Tarumã Açu neighborhood (2°58′54.0″S 60°02′49.0″W). Adults of *An. aquasalis* were obtained from the colony of Laboratory of infectious Ecology Diseases of Amazon facility of Leônidas e Maria Deane Institute (ILMD-Fiocruz AM).

### F1 mosquitoes

The field-collected females were blood-fed on chicken and on the fourth day, transferred to individual oviposition cups after a blood meal. Mosquito larvae collected in the field and those obtained through eclosion of hatching eggs were reared in the laboratory in plastic trays and fed Tetramin fish food. The emerged adults were maintained at 27 °C with 70% relative humidity and fed 10% sucrose solution *ad libitum*^48^. Mosquitoes were anesthetized at 4 °C and identified at the species level using the dichotomous key of Consoli and Oliveira^49^. They were maintained in cages at 27 °C with 70% relative humidity and fed 10% sucrose solution ad libitum.

### Blood collection and membrane-feeding assay

Blood from malaria-infected patients diagnosed by the thick blood smear method at the Dr. Heitor Vieira Dourado Tropical Medicine Foundation (FMT-HVD) was used for the experiments. The inclusion criteria were adult patients over 18 years old, infected with *P. vivax*, with two crosses of parasitemia (501–10,000 parasites/μl)^50^ who agreed to participate in the project as volunteers. For each patient, 5 mL of blood was collected in heparinized Vacutainer tubes.

Adult mosquitoes were starved for 24 h prior to blood feeding. The experimental infection was carried out through a membrane feeding assay using a hose system connected to a thermal bath held at 39 °C to keep the blood warm^14^. Oocysts in the midgut and sporozoites in the salivary glands of *An. aquasalis* were used as controls, and all species were fed with blood from the same malaria-infected patients*.* Fully engorged females were maintained at 27 °C with 70% relative humidity, and fed 10% sucrose solution ad libitum.

### Oocyst and sporozoite counts

Seven days post-infection, the midgut of mosquitoes was dissected in phosphate buffered saline (PBS; 1X) on glass slides, stained with 2% Mercurochrome (Merbromin), and observed under an optical microscope (Leica DM1000, Germany) at 40X magnification. The number of oocysts in each midgut sample was recorded. Fourteen days post-infection, the salivary glands of 20 mosquitoes were dissected in RPMI-1640, pooled, macerated, and sporozoites were counted using a hemocytometer slide under an optical light microscope at 40X magnification. The counts were performed according to the method described by Prinz et al.^51^ *An. aquasalis* oocysts and sporozoites were used as controls.

### Salivation

Salivation was performed on the day 14 post-infection. Twenty females of each species were held in saliva using a membrane feeding assay in glass feeders with 100 µL of uninfected blood for 2 h. The samples (blood and saliva) were transferred to microtubes and stored at − 20 °C.

### DNA extraction

Blood samples containing mosquito saliva were placed in 1.5 ml microtubes containing 1 ml distilled water, and DNA was extracted using the Chelex 100 Resin 5% (Bio-Rad) method. The samples were incubated at 27 °C for 30 min and vortexed for 15 s. After homogenization, the samples were centrifuged at 14,000 rpm for 3 min, the supernatant was removed, and 170 µl of 5% Chelex 100 Resin was added, heated in a thermoblock at 56 °C for 30 min, and vortexed for 15 s. Finally, the samples were incubated at 96 °C for 8 min, vortexed for 15 s, and centrifuged at 15,000 rpm for 3 min. The extracted DNA was stored at − 20 °C.

### Detection of *P. vivax*

PCR was performed using the GoTaq Flexi DNA Polymerase Kit (Promega, Madison, WI, USA) targeting a sequence of the Pv18S gene. The reaction was performed in a 25 μl final volume with 1 µL of DNA. The PCR cycle used is as follows: 10 min at 95 °C, 40 cycles of 15 s at 95 °C, and 1 min at 60 °C/1 cycle at 16 °C^52^. The samples were visualized via gel electrophoresis on a 2% agarose gel containing GelRed (Phenix Research). Gel was imaged with a UVP Epichemi^3^ Darkroom (UVP, LLC., Upland, CA).

### Ethical aspects

This study was approved by the Ethical Review Committee at FMT-HVD (CAAE 39706514.2.00000.0005), all subjects included in the study, or their legal guardians were informed about the project, and those accepted to participate assigned the informed consent terms. Field collection of mosquitoes was approved by Biodiversity Authorization and Information System (No. 75496). Animal Use Ethics Committee of National Institute of Amazonian Research—INPA (No. 008/2021, SEI 01280.000118/2021-34) approved the use of horses as attractive and chickens as blood meal resources for mosquitoes. All methods were carried out in accordance with relevant guidelines and regulations. This study is reported in accordance with ARRIVE guidelines.

### Statistical analysis

The infection rate (IR) was calculated by dividing the number of infected midguts by the number of dissected midguts and multiplying by 100. Oocyst infection intensity was calculated by dividing the total number of oocysts by the total number of infected mosquitoes. The mean number of sporozoites was calculated by dividing the total number of sporozoites by the total number of dissected female mosquitoes. The infection rate and intensity of all the mosquito species were compared with the infection rate and intensity of *An. aquasalis* (the reference species). The mean number of sporozoites in the salivary glands of all the mosquito species evaluated was compared using heatmaps. The Shapiro–Wilk test was used to test the normality of the data. The Kruskal–Wallis test was used to compare the number of oocysts and sporozoites between the evaluated species using the *An. aquasalis* as a reference species. Pearson’s correlation coefficient was calculated to evaluate the correlation between the infection intensity and sporozoite production. All data analyses were performed using the GraphPad Prism v.6 software (San Diego, CA, USA, https://www.graphpad.com/).

## Supplementary Information

Supplementary Information 1.

Supplementary Information 2.

Supplementary Video 1.

Supplementary Information 3.

## Data Availability

The datasets analyzed during the current study are available from the corresponding author on reasonable request. All methods were carried out in accordance with relevant guidelines and regulations.
