# Supplementary material for: Amazonian Anopheles with low numbers of oocysts transmit Plasmodium vivax sporozoites during a blood meal
Source: Sci Rep. 2022 Nov 14;12:19442. doi: 10.1038/s41598-022-24058-z (PMC9663451; doi:10.1038/s41598-022-24058-z)

Amazonian *Anopheles* with low numbers of oocysts transmit *Plasmodium vivax* sporozoites during a blood meal

Jordam William Pereira-Silva[1,3,4*], Keillen Monick Martins-Campos[1], José Vicente Ferreira-Neto[1,5], Marcus Vinicius Guimarães Lacerda[2,3,4], Felipe Arley Costa Pessoa[1,5] & Claudia María Ríos-Velásquez[1,4*]

1. Laboratório de Ecologia de Doenças Transmissíveis na Amazônia, Instituto Leônidas e Maria Deane, Fundação Oswaldo Cruz - FIOCRUZ, Manaus, Amazonas, Brasil.

2. Laboratório de Diagnóstico e Controle de Doenças Infecciosas na Amazônia, Instituto Leônidas e Maria Deane, Fundação Oswaldo Cruz - FIOCRUZ, Manaus, Amazonas, Brasil.

3. Fundação de Medicina Tropical Dr. Heitor Vieira Dourado, Manaus, Amazonas, Brasil.

4. Programa de Pós-graduação em Medicina Tropical, Escola Superior de Ciências da Saúde, Universidade do Estado do Amazonas, Manaus, Brasil.

5. Programa de Pós-Graduação em Entomologia, Instituto Nacional de Pesquisas da Amazônia, Manaus, Amazonas, Brasil.

*Corresponding Authors: Claudia María Ríos-Velásquez, Jordam William Pereira-Silva. Instituto Leônidas e Maria Deane - FIOCRUZ Amazônia, Laboratório de Ecologia de Doenças Transmissíveis na Amazônia, Rua Teresina, 476, Adrianópolis, Manaus, Amazonas, CEP: 69.057- 070, Brasil. E-mail: claudia.rios@fiocruz.br; jordamwilliam@gmail.com

Supplementary Figure 1. Gel electrophoresis image showing 100 bp band indicating amplified of *P. vivax* in the saliva of six species of *Anopheles*. (L) DNA Ladder; (1) *An. triannulatus* s.l.; (2) *An. nuneztovari* s.l.; (3) *An. benarrochi* s.l.; (4) *An. evansae*; (5) *An. darlingi*; (6) *An. aquasalis*; (7) Positive control (salivary gland + *P. vivax*); (8) Positive control (midgut + *P. vivax*); (9) Negative control.

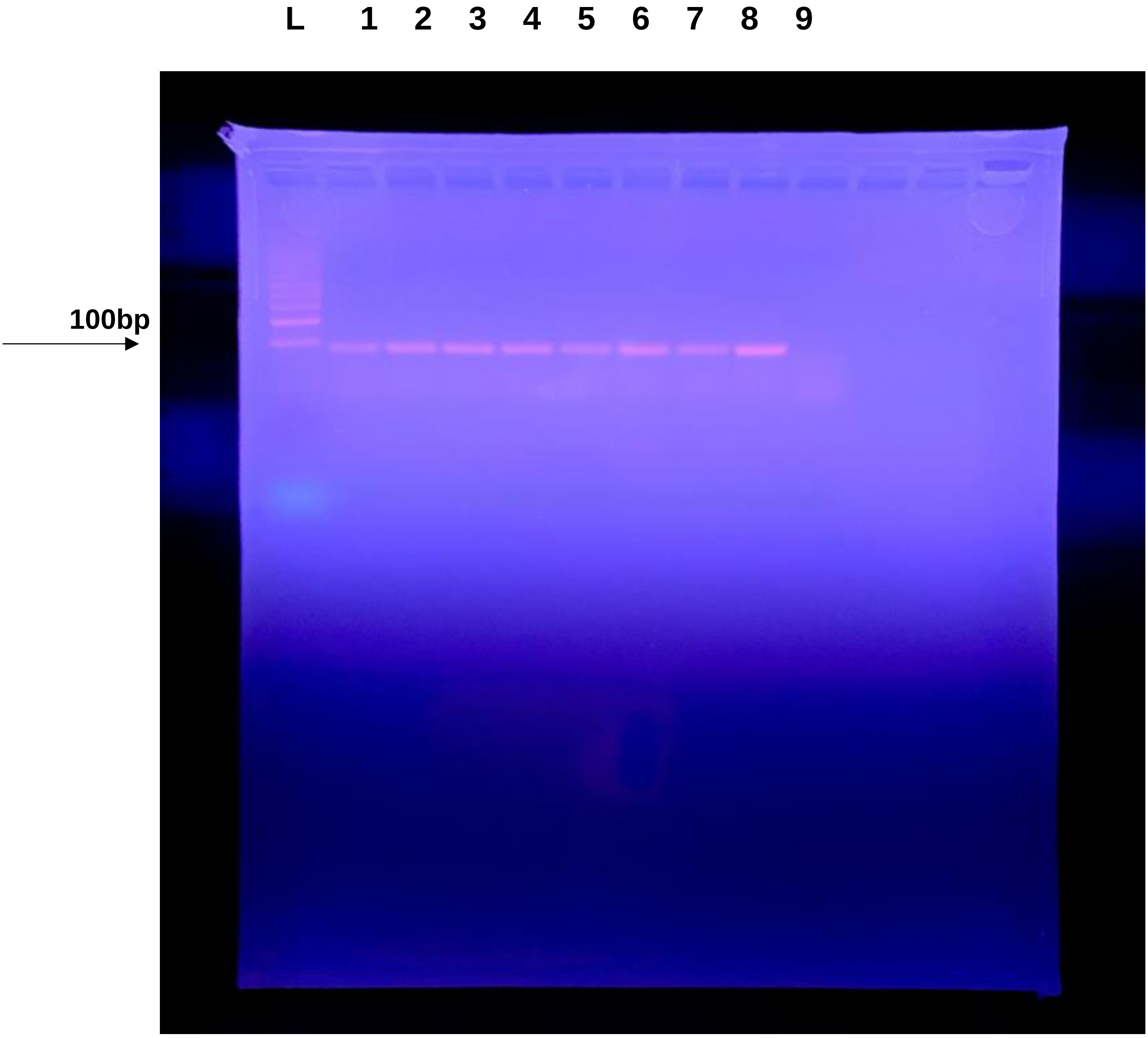

Supplement: Supplementary file 1 — Supplementary Information 1. [file 41598_2022_24058_MOESM1_ESM.pdf]
