# Supplementary material for: Amazonian Anopheles with low numbers of oocysts transmit Plasmodium vivax sporozoites during a blood meal
Source: Sci Rep. 2022 Nov 14;12:19442. doi: 10.1038/s41598-022-24058-z (PMC9663451; doi:10.1038/s41598-022-24058-z)
Supplement: Supplementary file 2 — Supplementary Information 2. [file 41598_2022_24058_MOESM2_ESM.docx]

**Additional information**

**Supplementary Table 1.** Susceptibility of six Amazonian *Anopheles* species fed with blood samples obtained from malaria-infected patients.

**Supplementary Movie 1.** *Plasmodium vivax* sporozoites in colonized *Anopheles aquasalis* mosquitoes in the Brazilian Amazon region. Optical microscope at 40 × magnification.

**Supplementary Figure 1.** Gel electrophoresis image showing 100 bp band indicating amplified of *P. vivax* in the saliva of six species of *Anopheles*. (L) DNA Ladder; (1) *An. triannulatus* s.l.; (2) *An. nuneztovari* s.l.; (3) *An. benarrochi* s.l.; (4) *An. evansae*; (5) *An. darlingi*; (6) *An. aquasalis*; (7) Positive control (salivary gland + *P. vivax*); (8) Positive control (midgut + *P. vivax*); (9) Negative control.
